# New 11,20-Epoxybriaranes from the Gorgonian Coral *Junceella fragilis* (Ellisellidae)

**DOI:** 10.3390/molecules24132487

**Published:** 2019-07-07

**Authors:** Chia-Cheng Lin, Jui-Hsin Su, Wu-Fu Chen, Zhi-Hong Wen, Bo-Rong Peng, Lin-Cyuan Huang, Tsong-Long Hwang, Ping-Jyun Sung

**Affiliations:** 1 Department of Neurosurgery, Kaohsiung Chang Gung Memorial Hospital and Chang Gung University College of Medicine, Kaohsiung 833, Taiwan; 2 National Museum of Marine Biology and Aquarium, Pingtung 944, Taiwan; 3 Graduate Institute of Marine Biology, National Dong Hwa University, Pingtung 944, Taiwan; 4 Department of Marine Biotechnology and Resources, National Sun Yat-sen University, Kaohsiung 804, Taiwan; 5 Research Center for Chinese Herbal Medicine, College of Human Ecology, Chang Gung University of Science and Technology, Taoyuan 333, Taiwan; 6 Research Center for Food and Cosmetic Safety, College of Human Ecology, Chang Gung University of Science and Technology, Taoyuan 333, Taiwan; 7 Graduate Institute of Healthy Industry Technology, College of Human Ecology, Chang Gung University of Science and Technology, Taoyuan 333, Taiwan; 8 Graduate Institute of Natural Products, College of Medicine, Chang Gung University, Taoyuan 333, Taiwan; 9 Chinese Herbal Medicine Research Team, Healthy Aging Research Center, Chang Gung University, Taoyuan 333, Taiwan; 10 Department of Anaesthesiology, Chang Gung Memorial Hospital, Taoyuan 333, Taiwan; 11 Graduate Institute of Natural Products, Kaohsiung Medical University, Kaohsiung 807, Taiwan; 12 Chinese Medicine Research and Development Center, China Medical University Hospital, Taichung 404, Taiwan

**Keywords:** *Junceella fragilis*, fragilide, briarane, superoxide anion

## Abstract

Two new 11,20-epoxybriaranes, fragilides P (**1**) and Q (**2**), as well as two known analogues, robustolide F (**3**) and juncin Z (**4**), were obtained from the gorgonian coral *Junceella fragilis*. The structures, including the absolute configurations of briaranes **1** and **2**, were elucidated by using spectroscopic methods and comparing the spectroscopic and rotation data with those of known related analogues. Briarane **4** decreased the generation of superoxide anions by human neutrophils. The propionate group in **1** is rarely found.

## 1. Introduction

**Figure 1:**
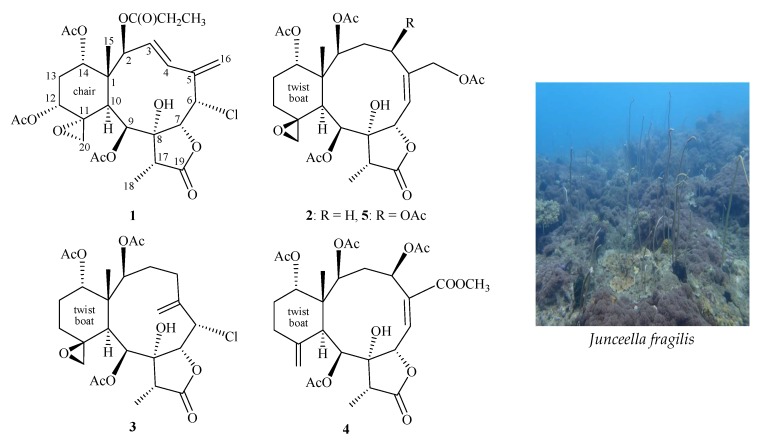
Structures of fragilides P (**1**), Q (**2**), juncins Z (**4**), X (**5**), and robustolide F (**3**) and a picture of *Junceella fragilis*.

Since the first structure elucidation of a briarane-type natural product, briarein A, in 1977 by single-crystal X-ray diffraction analysis [1], over 700 marine origin briaranes have been isolated and reported from various octocorals, especially from genera *Briareum* (family Briareidae) [2] and *Junceella* (family Ellisellidae) [3,4,5]. Among these compounds, 11,20-epoxybriaranes were proven to be a chemical marker for the gorgonian corals belonging to family Ellisellidae [6]. During the course of our research on new natural substances from the marine invertebrates distributed in the waters of Taiwan, a series of briarane-type diterpenoids were isolated from various octocorals belonging to the genera *Junceella* [7] and *Briareum* [8], and the compounds of this type were proven to possess various interesting bioactivities. Recently, we focused our ongoing studies on a gorgonian coral identified as *Junceella fragilis*. From the results of our studies on this species, we report herein the isolation, structural determination, and bioactivity of two new briaranes, fragilides P (**1**) and Q (**2**), along with two known metabolites, robustolide F (**3**) [9,10] and juncin Z (**4**) [11] (Figure 1).

## 2. Results and Discussion

Fragilide P (**1**) has the molecular formula C_29_H_37_ClO_12_ as deduced by (+)-ESIMS—which showed a pair of peaks at *m*/*z* 635/637 (3:1) [M + H^+^], suggesting a chlorine atom in **1**—and further confirmed by (+)-HRESIMS at *m*/*z* 635.18683 (calcd. for C_29_H_37_^35^ClO_12_ + Na, 635.18658). The IR spectrum of **1** indicated the presence of hydroxy (3466 cm^−1^), γ-lactone (1783 cm^−1^), and ester carbonyl (1735 cm^−1^) groups. The ^13^C-NMR spectral data (Table 1) showed the presence of a disubstituted olefin (δ_C_ 132.7, CH-4; 130.4, CH-3) and an exomethylene (δ_C_ 142.0, C-5; 115.1, CH_2_-16). Moreover, five carbonyl resonances at δ_C_ 174.6, 173.3, 170.3, 169.9, and 169.6 in the ^13^C spectrum confirmed the presence of a γ-lactone and four other ester groups. In the ^1^H NMR spectrum, three acetate methyls (δ_H_ 2.11, 2.09, 2.06, each 3H × s) and a propionate (δ_H_ 2.31, 2H, q, *J* = 7.6 Hz; 1.11, 3H, t, *J* = 7.6 Hz) were observed. An exocyclic epoxy group was elucidated from the signals of two oxygenated carbons at δ_C_ 57.3 (C-11) and 49.2 (CH_2_-20). The proton chemical shifts at δ_H_ 2.77 (1H, dd, *J* = 3.2, 1.2 Hz, H-20a) and 2.64 (1H, d, *J* = 3.2 Hz, H-20b) confirmed the presence of this group. Moreover, a methyl singlet, a methyl doublet, two aliphatic protons, a pair of aliphatic methylene protons, five oxymethine protons, a chlorinated methine proton, and a hydroxy proton were observed in the ^1^H-NMR spectrum of **1** (Table 1).

**Figure 2:**
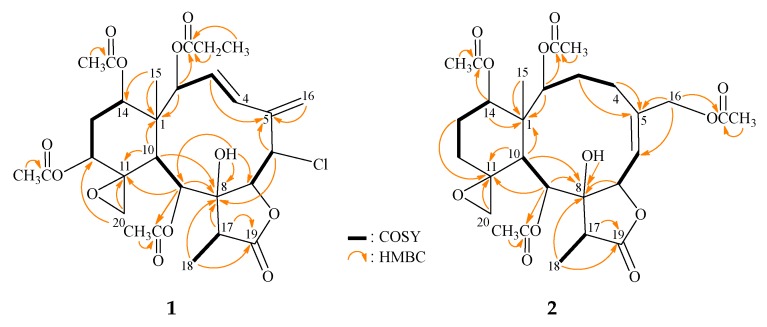
The COSY correlations and selective HMBC of **1** and **2**.

Analyses of 2D-NMR (COSY and Heteronuclear Multiple Bond Correlation (HMBC)) data established a tetracyclic nucleus. This assignment was evident from the spin systems from H-2 to H-3, H-3 to H-4, H-6 to H-7, H-9 to H-10, H-12 to H_2_-13, H_2_-13 to H-14, and H-17 to H_3_-18 (Figure 2), while the HMBC between protons and quaternary carbons, such as H-2, H-10, H_3_-15/C-1; H-3, H-6, H-16b/C-5; H-6, H-9, H-10, H-17, H_3_-18, OH-8/C-8; H-9, H-10, H-20b/C-11; and H-17, H_3_-18/C-19 revealed the carbon skeleton (Figure 2). The epoxy group positioned at C-11/20 was further confirmed by the HMBC between H-20b to C-11 and C-12. The C-15 methyl group was positioned at C-1 from the HMBC between H_3_-15 to C-1 and C-14. The HMBC spectrum also revealed that the carbon signal at δ_C_ 173.3 (C) was correlated with the signals of the methylene and methyl protons of propionate at δ_H_ 2.31 and 1.11, and it was assigned to the carbon atom of the propionate carbonyl group. The propionate at C-2 was confirmed from the connectivity between H-2 and the carbonyl carbon of the propionate group. The HMBC revealed that an acetoxy group is attached to C-9. The hydroxy group at C-8 was deduced from the HMBC of a hydroxy proton (δ_C_ 3.07) to C-7, C-8, and C-9. Thus, the remaining acetoxy groups were positioned at C-12 and C-14 by analysis of the characteristic NMR signals (δ_H_ 4.52, 1H, dd, *J* = 2.4, 2.4 Hz; δ_C_ 73.7, CH-12; δ_H_ 4.96, 1H, dd, *J* = 2.4, 2.4 Hz; δ_C_ 73.1, CH-14), although no HMBC was observed between H-12 and H-14 and the acetate carbonyl carbons.

**Figure 3:**
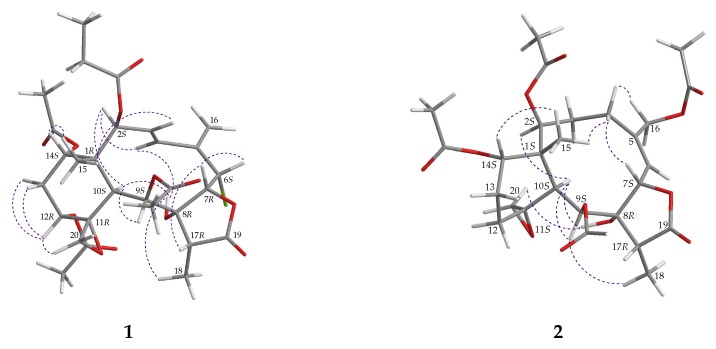
Selected protons with key NOESY (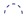) correlations of **1** and **2**.

According to a summary of the chemical shifts of 11,20-epoxy groups in briarane derivatives, with ^13^C-NMR data for C-11 and C-20 at δ_C_ 55−61 and 47−52 ppm, respectively, the epoxy group was α-oriented and the cyclohexane ring existed in a chair conformation [12]; hence, the configuration of the 11,20-epoxy group in **1** (δ_C_ 57.3, C-11; 49.2, CH_2_-20) should be α-oriented, and the cyclohexane ring should be in a chair conformation. The *E* configuration of the C-3/4 double bond was determined from the large proton coupling constant (*J* = 15.6 Hz) between H-3 and H-4. The stereochemistry of the 11 stereogenic centers of **1** was established by analysis of NOE correlations observed in a NOESY experiment and further supported by molecular mechanics 2 (MM2) force field analysis [13], as shown in Figure 3. In the NOESY spectrum, NOE correlations were observed between H-10 and H-2/H-9/OH-8, while no NOE correlation was seen with Me-15, suggesting that H-2, H-9, H-10, and OH-8 were all α-oriented; meanwhile, a NOE correlation of Me-15 with H-14 indicated that H-14 was β-oriented. In addition, H-12 was found to correlate with H-13α/β and one proton of C-20 methylene (δ_H_ 2.77, H-20a), indicating that the C-12 acetoxy group was α-oriented. H_3_-18 showed a NOE correlation with OH-8, indicating that Me-18 was α-oriented at C-17. H-7 exhibited NOE correlations with H-6 and H-17, suggesting that H-6 and H-7 were positioned on the β face. Furthermore, H-3 showed a NOE correlation with H_3_-15; and H-4 showed NOE correlations with H-2 and OH-8, demonstrating the *E*-configuration of Δ^3^ and establishing the *s-cis* diene moiety. As briaranes **1** and **2** were isolated along with a known metabolite **3** (robustolide F) from the same organism, and the absolute configuration of **3** was determined by single-crystal X-ray diffraction analysis [10], it is reasonable on biogenetic grounds and supported by the equal sign of optical rotation of **1**, **2**, and **3** to assume that **1** and **2** have the same absolute configurations as **3**. Therefore, based on the above findings, the configurations of the stereogenic carbons of **1** were determined as 1*R*, 2*S*, 6*S*, 7*R*, 8*R*, 9*S*, 10*S*, 11*R*, 12*R*, 14*S*, and 17*R* (see Figures S1–S10). It is interesting to note that the propionate group is rarely found in briarane-type natural products [12,14,15,16,17,18].

Fragilide Q (**2**) was found to have the molecular formula C_2__8_H_3__8_O_1__2_ as determined from its (+)-HRESIMS at *m*/*z* 589.22562 (calcd. for C_2__8_H_3__8_O_1__2_ + Na, 589.22555) (Ω = 10). Its absorption peaks in the IR spectrum showed ester carbonyl, γ-lactone, and broad OH stretching at 1740, 1778, and 3273 cm^−1^, respectively. It was found that the ^1^H and ^13^C-NMR spectra of **2** resembled those of a known analogue, juncin X (**5**) (Figure 1), isolated from gorgonian coral *Junceella* *juncea* collected off the South China Sea [11], except that the signals corresponding to the acetoxy group at C-4 in **5** were replaced by a proton in **2**. The locations of the functional groups were further confirmed by HMBC and COSY correlations (Figure 2); hence, fragilide Q was assigned the structure of **2**, with the same stereochemistry as that of **1**, and the configurations of the stereogenic carbons were elucidated as 1*S*, 2*S*, 7*S*, 8*R*, 9*S*, 10*S*, 11*S*, 14*S*, and 17*R* (Figure 3) (see Figures S11–S20). Due to the chemical shifts for C-11 and C-20 which appeared at δ_C_ 62.4 and 59.0 ppm, respectively, the epoxy group was β-oriented and the cyclohexane ring should exist in a twisted boat conformation [12].

Two known briaranes were isolated and identified as robustolide F (**3**) [9,10] and juncin Z (**4**) [11] by way of comparison with the spectroscopic and physical data reported in the literature.

In an in vitro anti-inflammatory activity assay, it was found that briarane **4** (juncin Z) showed a 25.56% inhibitory effect on the generation of superoxide anions by human neutrophils at a concentration of 10 μM, and briaranes **1**–**3** were inactive.

## 3. Materials and Methods

### 3.1. General Experimental Procedures

The optical rotations were recored using a Jasco P-1010 digital polarimeter (Japan Spectroscopic, Tokyo, Japan). IR spectra were measured on a Thermo Scientific Nicolet iS5 FT-IR spectrophotometer (Waltham, MA, USA). NMR spectra were taken on a Jeol Resonance ECZ 400S (Tokyo, Japan) or on a Varian Inova (Palo, Alto, CA, USA) 500 NMR spectrometer using the residual CHCl_3_ signal (δ_H_ 7.26 ppm) and CDCl_3_ (δ_C_ 77.1 ppm) as the internal standard for ^1^H and ^13^C-NMR, respectively; coupling constants (J) are presented in Hz. Multiplicities of ^13^C-NMR data were determined by Distortionless Enhancement by Polarization Transfer (DEPT) experiments. ESIMS and HRESIMS mass spectra were measured on a Bruker mass spectrometer with 7 tesla magnets (model: SolariX FTMS system; Bruker, Bremen, Germany). HPLC separations were carried out on a Hitachi L-2130 pump (Tokyo, Japan) equipped with a Hitachi L-2455 photodiode array detector. The column used for HPLC was reversed-phase silica (250 mm × 21.2 mm, 5 μM, Luna RP-18e; Phenomenex Inc., Torrance, CA, USA). Column chromatography was carried out with Kieselgel 60 (230–400 mesh, Merck, Darmstadt, Germany). TLC was performed on precoated Kieselgel 60 F_254_ (0.25 mm thick, Merck), then sprayed with 10% H_2_SO_4_ solution, followed by heating to visualize the spots.

### 3.2. Animal Material

The sea whip gorgonian coral *Junceella fragilis* was collected by hand in April 2017 using self-contained underwater breathing apparatus (SCUBA) gear at depths of 10–15 m off the coast of South Bay, Kenting, Taiwan. The samples were then stored in a −20 °C freezer until extraction. A voucher specimen was deposited in the National Museum of Marine Biology and Aquarium, Taiwan (NMMBA-TW-GC-2017-022). Identification of the species of this organism was performed by comparison as described in previous publications [3,4,5].

### 3.3. Extraction and Isolation

The freeze-dried and sliced bodies (wet/dry weight = 795/313 g) of the coral specimen were prepared and extracted with a 1:1 mixture of MeOH and CH_2_Cl_2_ to give 19.0 g of crude extract which was partitioned between EtOAc and H_2_O. The EtOAc extract (8.0 g) was applied on silica gel column chromatography (C.C.) and eluted with gradients of n-hexane/acetone (50:1 to 1:2, stepwise) to furnish eight fractions (fractions A−H). Fraction G was chromatographed on silica gel C.C. and eluted with gradients of n-hexane/EtOAc (4:1 to 1:1, stepwise) to afford 16 subfractions (fractions G1−G16). Afterward, fraction G9 was separated by RP-HPLC using a mixture of MeOH and H_2_O (with volume/volume = 60:40; at a flow rate of 4.0 mL/min) to afford fragilide P (**1**, 2.7 mg), fragilide Q (**2**, 1.8 mg), robustolide F (**3**, 1.4 mg), and juncin Z (**4**, 1.2 mg).

Fragilide P (**1**): amorphous powder; [α${]}_{D}^{27}$ −14 (c 0.9, CHCl_3_); IR (ATR) ν_max_ 3466, 1783, 1735 cm^−1^; ^1^H and ^13^C-NMR data (see Table 1); ESIMS: m/z 635 [M + Na]^+^; HRESIMS: m/z 635.18683 (calcd. for C_29_H_37_^35^ClO_12_ + Na, 635.18658).

Fragilide Q (**2**)**:** amorphous powder; [α${]}_{D}^{28}$ −59 (*c* 0.6, CHCl_3_); IR (ATR) ν_max_ 3273, 1778, 1740 cm^−1^; ^1^H and ^13^C-NMR data (see Table 1); ESIMS: *m*/*z* 589 [M + Na]^+^; HRESIMS: *m*/*z* 589.22562 (calcd. for C_28_H_38_O_12_ + Na, 589.22555).

Robustolide F (**3**): amorphous powder; [α${]}_{D}^{23}$ −37 (*c* 0.07, CHCl_3_) (ref. [9] [α${]}_{D}^{26}$ −26.8 (*c* 1.038, CHCl_3_)); ref. [10] [α${]}_{D}^{25}$ −28 (*c* 0.24, CHCl_3_)); IR (ATR) ν_max_ 3288, 1780, 1735 cm^−1^; ^1^H and ^13^C-NMR data were found to be in absolute agreement with previous studies [9]; ESIMS: *m*/*z* 565 [M + Na]^+^.

Juncin Z (**4**): amorphous powder; [α${]}_{D}^{23}$ +28 (*c* 0.06, CHCl_3_) (ref. [11] [α]D +31.57 (*c* 0.95, CHCl_3_)); IR (ATR) ν_max_ 3433, 1782, 1738 cm^−1^; ^1^H and ^13^C-NMR data were found to be in absolute agreement with previous studies [11]; ESIMS: *m*/*z* 617 [M + Na]^+^.

### 3.4. Molecular Mechanics Calculations

The molecular models were generated by implementing the MM2 force field [13] in ChemBio 3D Ultra software (version 12.0) which was created by CambridgeSoft (PerkinElmer, Cambridge, MA, USA).

### 3.5. Superoxide Anion Generation by Human Neutrophils 

Human neutrophils were obtained by means of dextran sedimentation and Ficoll centrifugation. Measurements of elastase release and superoxide anion generation were carried out according to previously described procedures [19]. Briefly, superoxide anion production was assayed by monitoring the superoxide-dismutase-inhibitable reduction of ferricytochrome c. Elastase release experiments were performed using MeO-Suc-Ala-Ala-Pro-Valp-nitroanilide as the elastase substrate.

## 4. Conclusions

The sea whip gorgonian coral *Junceella fragilis*, a zooxanthella-containing species [20], has been demonstrated to have a wide structural diversity of interesting marine-origin briarane-type diterpenoids [7], and the compounds of this type were suggested originally to be produced by the host corals and not by its zooxanthellae [21]. In our continued study of *Junceella fragilis* collected in the waters of Taiwan, two previously unreported briaranes, fragilides P (**1**) and Q (**2**), were isolated along with two previously described analogues, robustolide F (**3**) and juncin Z (**4**). The structures, including the absolute configurations of **1** and **2**, were determined by using spectroscopic methods and comparing the spectroscopic and rotation values with those of a known related analogue, robustolide F (**3**) [9,10]. Juncin Z (**4**) was found to display an inhibitory effect on the generation of superoxide anions by human neutrophils.

## Figures and Tables

**Table 1:** ^1^H and ^13^C-NMR data for **1** and **2**.

	1		2	
C/H	δ_H_^a^ (*J* in Hz)	δ_C_, ^b^ Mult.	δ_H_^c^ (*J* in Hz)	δ_C_, ^d^ Mult.
1		49.2, C		46.9, C
2	5.73 d (9.6)	75.6, CH	4.80 d (5.0)	74.8, CH
3	6.00 dd (15.6, 9.6)	130.4, CH	2.44 m; 1.68 m	32.3, CH_2_
4	6.89 d (15.6)	132.7, CH	2.47 m; 2.01 m	24.9, CH_2_
5		142.0, C		139.8, C
6	5.07 d (4.0)	65.0, CH	5.53 d (10.5)	119.2, CH
7	4.16 d (4.0)	80.6, CH	5.20 d (10.5)	77.0, CH
8		82.8, C		80.4, C
9	5.19 d (2.0)	72.2, CH	5.61 d (6.0)	67.6, CH
10	3.84 br s	33.8, CH	2.29 d (6.0)	39.6, CH
11		57.3, C		62.4, C
12	4.52 dd (2.4, 2.4)	73.7, CH	2.28 m; 1.16 m	23.6, CH_2_
13α/β	2.32 m; 2.06 m	29.0, CH_2_	1.77 ddd (15.5, 10.0, 10.0); 2.16 m	24.5, CH_2_
14	4.96 dd (2.4, 2.4)	73.1, CH	4.90 d (5.0)	72.9, CH
15	1.18 s	14.4, CH_3_	1.01 s	14.9, CH_3_
16a/b	5.34 s; 5.26 s	115.1, CH_2_	5.26 dd (16.0, 2.0); 4.23 d (16.0)	67.1, CH_2_
17	2.84 q (7.2)	50.1, CH	2.34 q (7.0)	42.3, CH
18	1.25 d (7.2)	6.9, CH_3_	1.16 d (7.0)	6.7, CH_3_
19		174.6, C		176.4, C
20a/b	2.77 dd (3.2, 1.2); 2.64 d (3.2)	49.2, CH_2_	2.82 d (4.5); 3.23 br d (4.5)	59.0, CH_2_
2-OCOEt	2.31 q (7.6) 1.11 t (7.6)	173.3, C 27.7, CH_2_ 8.8, CH_3_		
Acetate methyls	2.11 s 2.09 s 2.06 s	21.4, CH_3_ 21.1, CH_3_ 21.0, CH_3_	2.22 s 2.14 s 2.04 s 2.01 s	21.0, CH_3_ 20.9, CH_3_ 20.9, CH_3_ 20.9, CH_3_
Acetate carbonyls		170.3, C 169.9, C 169.6, C		170.8, C 170.8, C 170.2, C 169.5, C
8-OH	3.07 s		5.19 s	

^a^ Spectra recorded at 400 MHz in CDCl_3_ at 25 °C. ^b^ Spectra recorded at 100 MHz in CDCl_3_ at 25 °C. ^c^ Spectra recorded at 500 MHz in CDCl_3_ at 25 °C. ^d^ Spectra recorded at 125 MHz in CDCl_3_ at 25 °C.

## Supplementary Materials

The Supplementary Materials are available online. ESIMS, HRESIMS, IR, 1D (^1^H, ^13^C-NMR, and DEPT spectra) and 2D (HSQC, COSY, HMBC, and NOESY) spectra of new compounds **1** and **2** and ESIMS, ^1^H, ^13^C-NMR, and DEPT spectra of **3** and **4**.

## References

[1] Burks J.E., van der Helm D., Chang C.Y., Ciereszko L.S. (1977). The crystal and molecular structure of briarein A, a diterpenoid from the gorgonian *Briareum asbestinum*. Acta Cryst..

[2] Samimi-Namin K., van Ofwegen L.P. (2016). Overview of the genus *Briareum* (Cnidaria, Octocorallia, Briareidae) in the Indo-Pacific, with the description of a new species. ZooKeys.

[3] Bayer F.M. (1981). Key to the genera of octocorallia of Pennatulacea (Coelenterata: Anthozoa), with diagnoses of new taxa. Proc. Biol. Soc. Wash..

[4] Bayer F.M., Grasshoff M. (1994). The genus group taxa of the family Ellisellidae, with clarification of the genera established by J.E. Gary (Cnidaria: Octocorallia). Senckenberg. Biol..

[5] Chen C.-C., Chang K.-H. (1991). Gorgonacea (Coelenterata: Octocorallia) of Southern Taiwan. Bull. Inst. Zool. Acad. Sin..

[6] Su Y.-M., Fan T.-Y., Sung P.-J. (2007). 11,20-Epoxybriaranes from the gorgonian coral *Ellisella robusta* (Ellisellidae). Nat. Prod. Res..

[7] Chung H.-M., Wang Y.-C., Tseng C.-C., Chen N.-F., Wen Z.-H., Fang L.-S., Hwang T.-L., Wu Y.-C., Sung P.-J. (2018). Natural product chemistry of gorgonian corals of genus *Junceella*–Part III. Mar. Drugs.

[8] Su Y.-D., Su J.-H., Hwang T.-L., Wen Z.-H., Sheu J.-H., Wu Y.-C., Sung P.-J. (2017). Briarane diterpenoids isolated from octocorals between 2014 and 2016. Mar. Drugs.

[9] Tanaka C., Yamamoto Y., Otsuka M., Tanaka J., Ichiba T., Marriott G., Rachmat R., Higa T. (2004). Briarane diterpenes from two species of octocorals, *Ellisella* sp. and *Pteroeides* sp.. J. Nat. Prod..

[10] Sung P.-J., Chiang M.Y., Tsai W.-T., Su J.-H., Su Y.-M., Wu Y.-C. (2007). Chlorinated briarane-type diterpenoids from the gorgonian coral *Ellisella robusta* (Ellisellidae). Tetrahedron.

[11] Qi S.-H., Zhang S., Qian P.-Y., Xiao Z.-H., Li M.-Y. (2006). Ten new antifouling briarane diterpenoids from the South China Sea gorgonian *Junceella juncea*. Tetrahedron.

[12] Sheu J.-H., Chen Y.-P., Hwang T.-L., Chiang M.Y., Fang L.-S., Sung P.-J. (2006). Junceellolides J–L, 11,20- epoxybriaranes from the gorgonian coral *Junceella fragilis*. J. Nat. Prod..

[13] Allinger N.L. (1977). Conformational analysis. 130. MM2. A hydrocarbon force field utilizing *V*_1_ and *V*_2_ torsional terms. J. Am. Chem. Soc..

[14] Guerriero A., D’Ambrosio M., Pietra F. (1995). Bis-allylic reactivity of the funicolides, 5,8(17)-diunsaturated briarane diterpenes of the sea pen *Funiculina quadrangularis* from the Tuscan archipelago, leading to 16-nortaxane derivatives. Helv. Chim. Acta.

[15] Chiasera G., Guerriero A., D’Ambrosio M., Pietra F. (1995). On the funicolides, briaranes of the Pennatulacean coral *Funiculina quadrangularis* from the Tuscan archipelago: Conformational preferences in this class of diterpenes. Helv. Chim. Acta.

[16] Sheu J.-H., Sung P.-J., Su J.-H., Wang G.-H., Duh C.-Y., Shen Y.-C., Chiang M.Y., Chen I.-T. (1999). Excavatolides U–Z, new briarane diterpenes from the gorgonian *Briareum excavatum*. J. Nat. Prod..

[17] Sung P.-J., Chen Y.-P., Su Y.-M., Hwang T.-L., Hu W.-P., Fan T.-Y., Wang W.-H. (2007). Fragilide B: A novel briarane-type diterpenoid with a *s-cis* diene moiety. Bull. Chem. Soc. Jpn..

[18] Cheng W., Ji M., Li X., Ren J., Yin F., van Ofwegen L., Yu S., Chen X., Lin W. (2017). Fragilolides A–Q, norditerpenoid and briarane diterpenoids from the gorgonian coral *Junceella fragilis*. Tetrahedron.

[19] Yu H.-P., Hsieh P.-W., Chang Y.-J., Chung P.-J., Kuo L.-M., Hwang T.-L. (2011). 2-(2-Fluorobenzamido) benzoate ethyl ester (EFB-1) inhibits superoxide production by human neutrophils and attenuates hemorrhagic shock-induced organ dysfunction in rats. Free Radic. Biol. Med..

[20] Walker T.A., Bull G.D. (1983). A newly discovered method of reproduction in gorgonian coral. Mar. Ecol. Prog. Ser..

[21] Kokke W.C.M.C., Epstein S., Look S.A., Rau G.H., Fenical W., Djerassi C. (1984). On the origin of terpenes in symbiotic associations between marine invertebrates and algae (zooxanthellae). J. Biol. Chem..

