# Supplementary material for: New 11,20-Epoxybriaranes from the Gorgonian Coral Junceella fragilis (Ellisellidae)
Source: Molecules. 2019 Jul 7;24(13):2487. doi: 10.3390/molecules24132487 (PMC6659381; doi:10.3390/molecules24132487)
Supplement: Supplementary file 1 [file molecules-24-02487-s001.pdf]

Supporting material

New 11,20-Epoxybriaranes from the Gorgonian Coral *Junceella fragilis* (Ellisellidae)

Chia-Cheng Lin [1], Jui-Hsin Su [2,3], Wu-Fu Chen [1,4], Zhi-Hong Wen [4], Bo-Rong Peng [2], Lin-Cyuan Huang [2,3,*], Tsong-Long Hwang [5,6,7,8,9,10,*] and Ping-Jyun Sung [2,3,4,11,12,*]

1 Department of Neurosurgery, Kaohsiung Chang Gung Memorial Hospital and Chang Gung University College of Medicine, Kaohsiung 833, Taiwan; dives123456@cgmh.org.tw (C.C.-L.); ma4949@adm.cgmh.org.tw (W.-F.C.)
2 National Museum of Marine Biology and Aquarium, Pingtung 944, Taiwan; x2219@nmmba.gov.tw (J.-H.S.); pengpojung@gmail.com (B.-R.P.)
3 Graduate Institute of Marine Biology, National Dong Hwa University, Pingtung 944, Taiwan
4 Department of Marine Biotechnology and Resources, National Sun Yat-sen University, Kaohsiung 804, Taiwan; wzh@mail.nsysu.edu.tw
5 Research Center for Chinese Herbal Medicine, College of Human Ecology, Chang Gung University of Science and Technology, Taoyuan 333, Taiwan
6 Research Center for Food and Cosmetic Safety, College of Human Ecology, Chang Gung University of Science and Technology, Taoyuan 333, Taiwan
7 Graduate Institute of Healthy Industry Technology, College of Human Ecology, Chang Gung University of Science and Technology, Taoyuan 333, Taiwan
8 Graduate Institute of Natural Products, College of Medicine, Chang Gung University, Taoyuan 333, Taiwan
9 Chinese Herbal Medicine Research Team, Healthy Aging Research Center, Chang Gung University, Taoyuan 333, Taiwan
10 Department of Anaesthesiology, Chang Gung Memorial Hospital, Taoyuan 333, Taiwan
11 Graduate Institute of Natural Products, Kaohsiung Medical University, Kaohsiung 807, Taiwan
12 Chinese Medicine Research and Development Center, China Medical University Hospital, Taichung 404, Taiwan
* Correspondence: bigpa830123@gmail.com (L.-C.H.); htl@mail.cgu.edu.tw (T.-L.H.); pjsung@nmmba.gov.tw (P.-J.S.); Tel.: +886-8-882-5001 (ext. 1384) (L.-C.H.); +886-3-211-8800 (ext. 5523) (T.-L.H.); +886-8-882-5037 (P.-J.S.); Fax: +886-8-882-5087 (L.-C.H. & P.-J.S.); +886-3-211-8506 (T.-L.H.).

S1. ESIMS spectrum of compound **1** 3
S2. HRESIMS spectrum of compound **1** 3
S3. IR spectrum of compound **1** 4
S4. ^{1}H NMR spectrum (400 MHz) of compound **1** in $CDCl_3$ 4
S5. ^{13}C NMR spectrum (100 MHz) of compound **1** in $CDCl_3$ 5
S6. DEPT spectrum of compound **1** in $CDCl_3$ 5
S7. HSQC spectrum of compound **1** in $CDCl_3$ 6
S8. HMBC spectrum of compound **1** in $CDCl_3$ 6
S9. ^{1}H-^{1}H COSY spectrum of compound **1** in $CDCl_3$ 7
S10. NOESY spectrum of compound **1** in $CDCl_3$ 7
S11. ESIMS spectrum of compound **2** 8
S12. HRESIMS spectrum of compound **2** 8
S13. IR spectrum of compound **2** 9
S14. ^{1}H NMR spectrum (500 MHz) of compound **2** in $CDCl_3$ 9
S15. ^{13}C NMR spectrum (125 MHz) of compound **2** in $CDCl_3$ 10
S16. DEPT spectrum (125 MHz) of compound **2** in $CDCl_3$ 10
S17. HSQC spectrum of compound **2** in $CDCl_3$ 11
S18. HMBC spectrum of compound **2** in $CDCl_3$ 11
S19. ^{1}H-^{1}H COSY spectrum of compound **2** in $CDCl_3$ 12
S20. NOESY spectrum of compound **2** in $CDCl_3$ 12
S21. ESIMS spectrum of compound **3** 13
S22. ^{1}H NMR spectrum (400 MHz) of compound **3** in $CDCl_3$ 13
S23. ^{13}C NMR spectrum (100 MHz) of compound **3** in $CDCl_3$ 14
S24. DEPT spectrum (100 MHz) of compound **3** in $CDCl_3$ 14
S25. ESIMS spectrum of compound **4** 15
S26. ^{1}H NMR spectrum (400 MHz) of compound **4** in $CDCl_3$ 15
S27. ^{13}C NMR spectrum (100 MHz) of compound **4** in $CDCl_3$ 16
S28. DEPT spectrum (100 MHz) of compound **4** in $CDCl_3$ 16

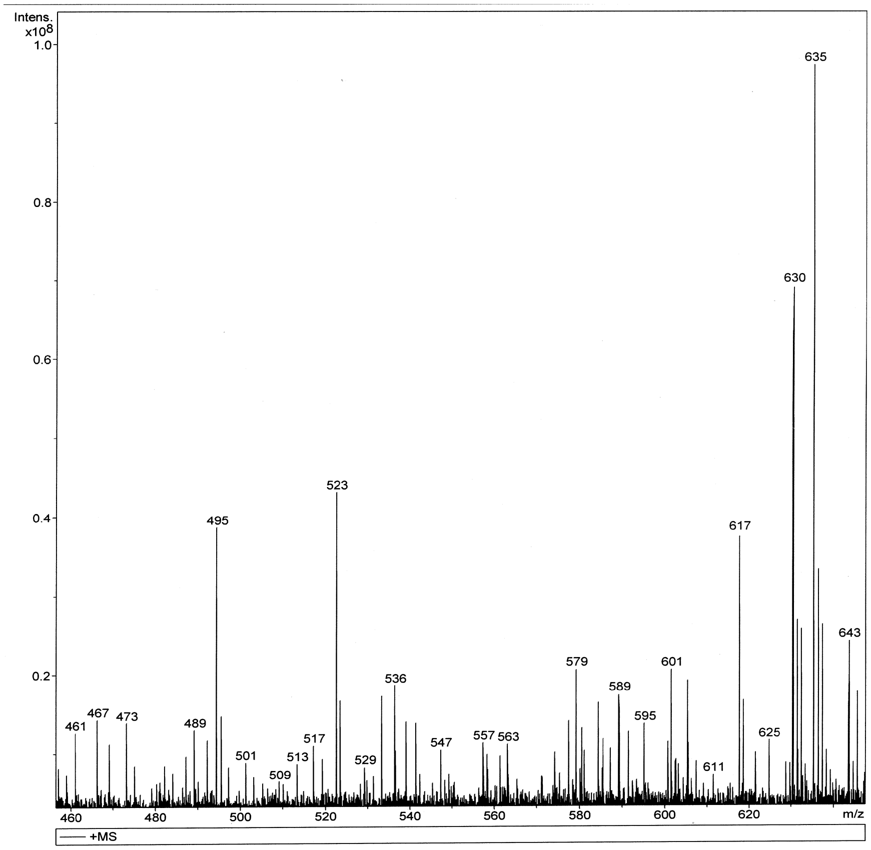

S1. ESIMS spectrum of compound **1**

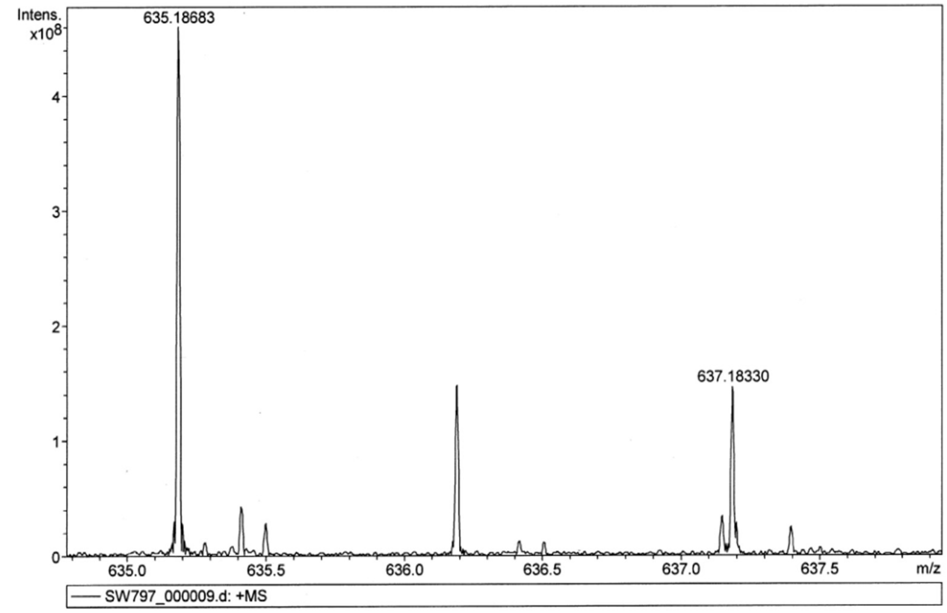

Meas. m/z	#	Formula	Score	m/z	err [mDa]	err [ppm]	mSigma	rdb	e⁻ Conf	N-Rule
635.18683	1	C 29 H 37 Cl Na O 12	100.00	635.18658	-0.25	-0.40	36.3	10.5	even	ok

S2. HRESIMS spectrum of compound **1**

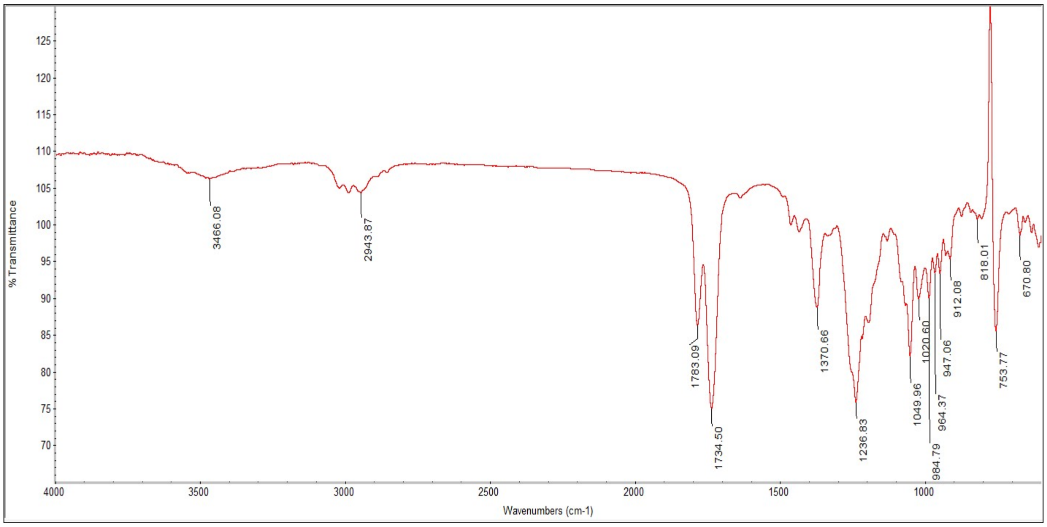

S3. IR spectrum of compound **1**

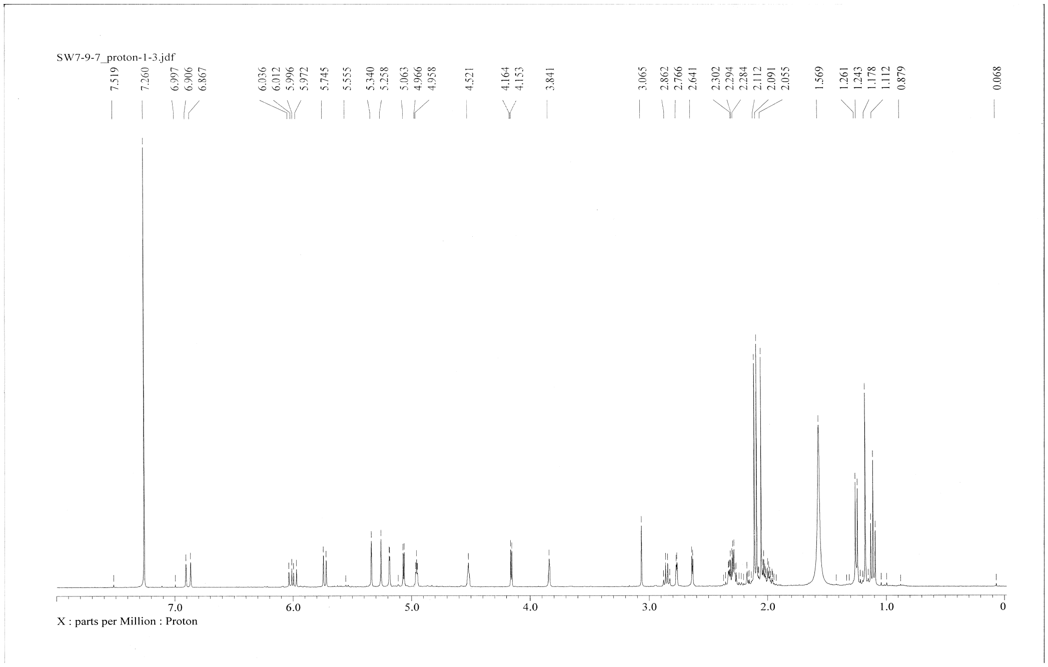

S4. ^{1}H NMR spectrum (400 MHz) of compound **1** in $CDCl_3$

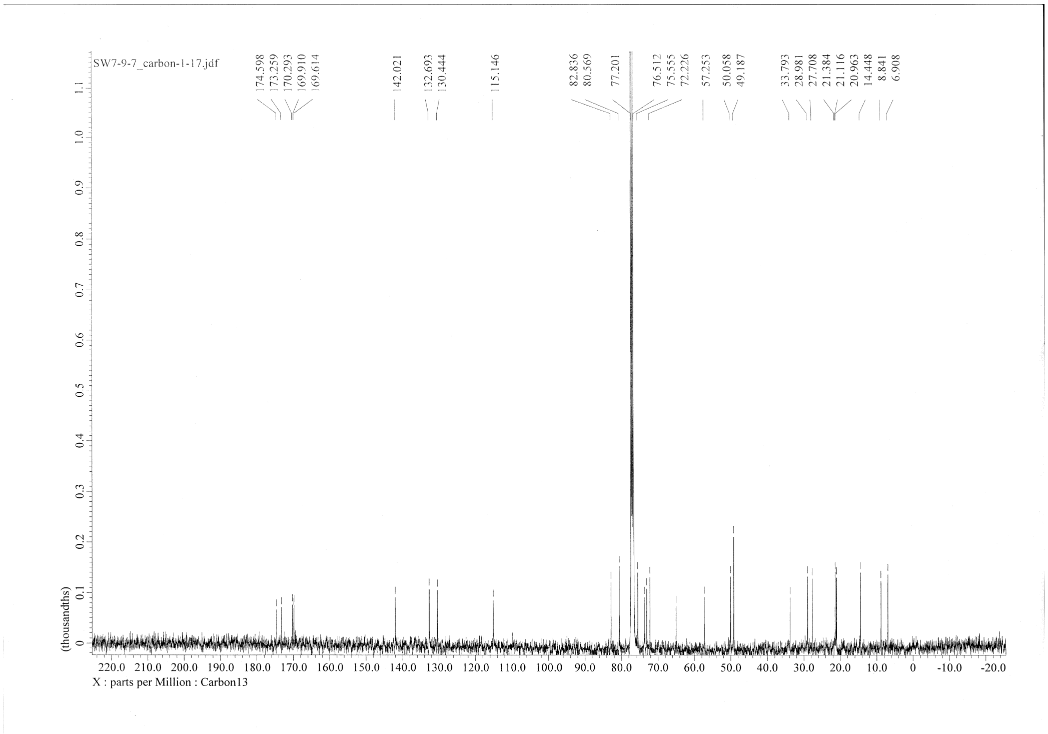

S5. ^{13}C NMR spectrum (100 MHz) of compound **1** in $CDCl_3$

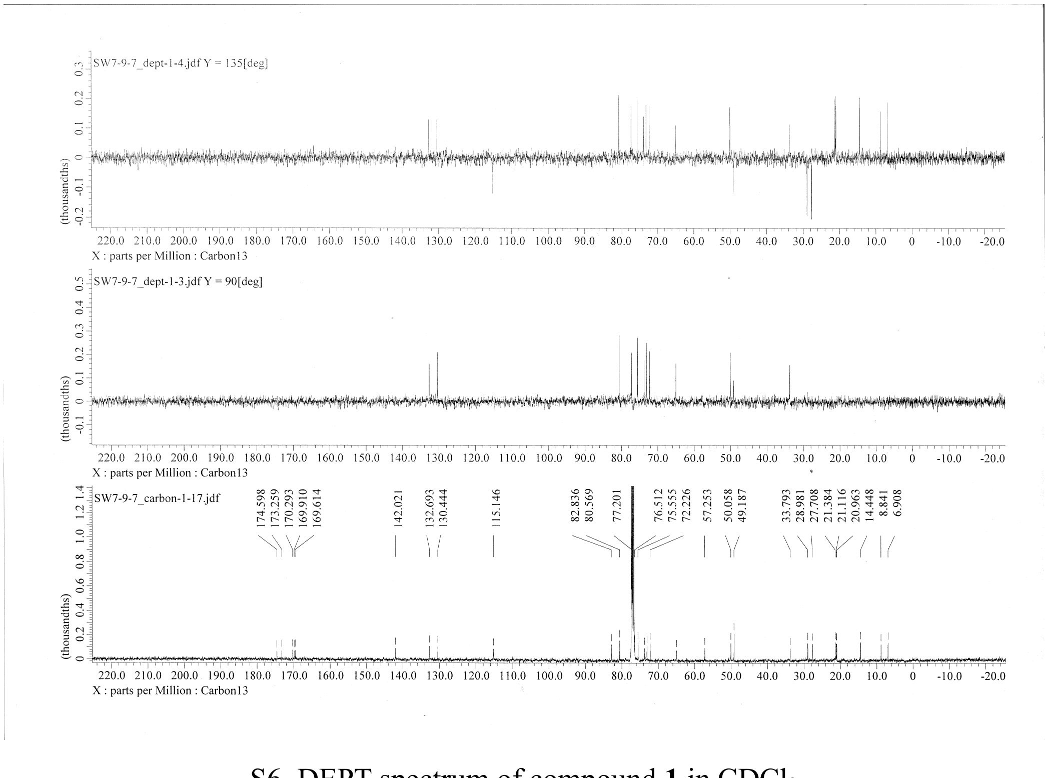

S6. DEPT spectrum of compound **1** in $CDCl_3$

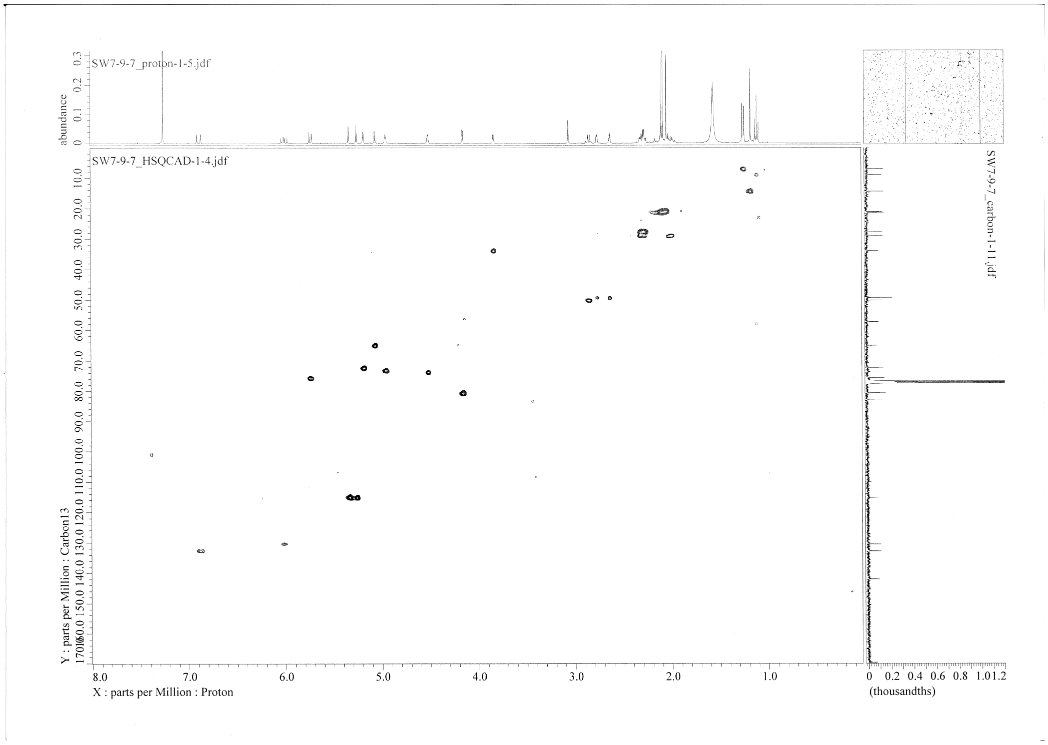

S7. HSQC spectrum of compound **1** in $CDCl_3$

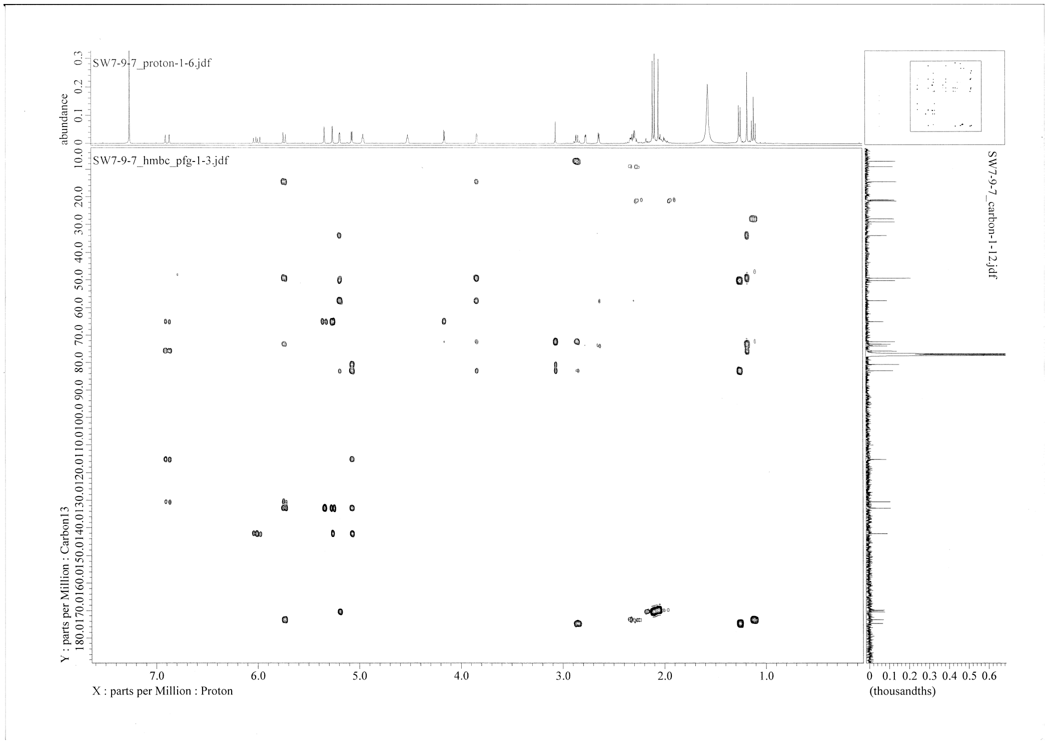

S8. HMBC spectrum of compound **1** in $CDCl_3$

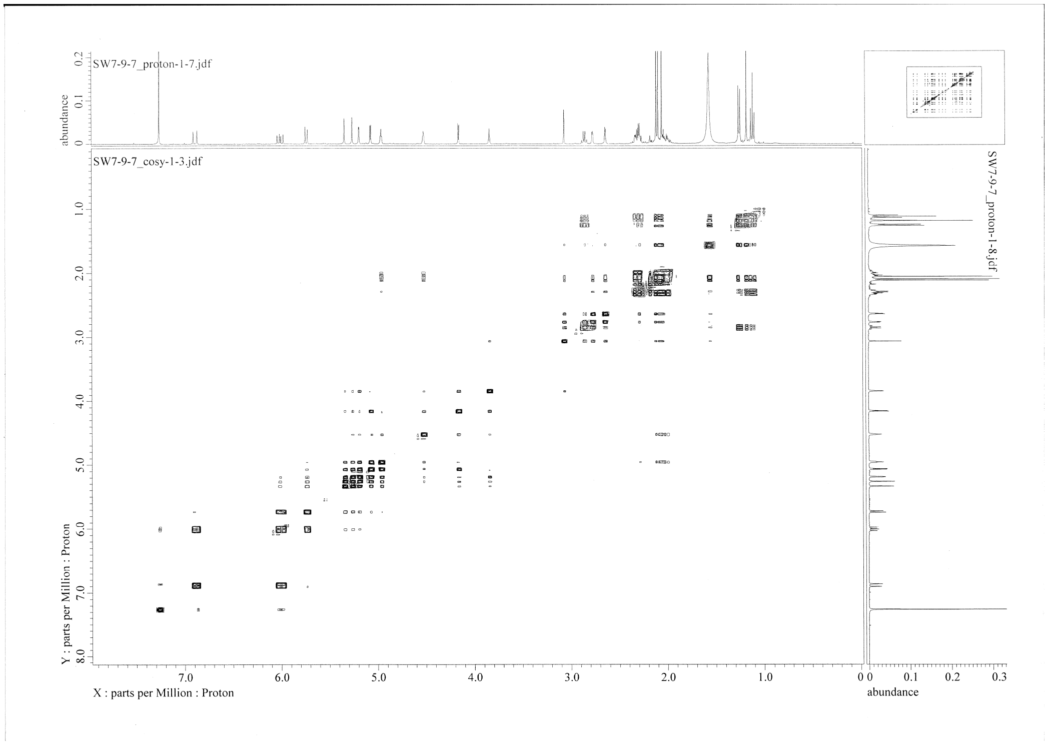

S9. COSY spectrum of compound **1** in $CDCl_3$

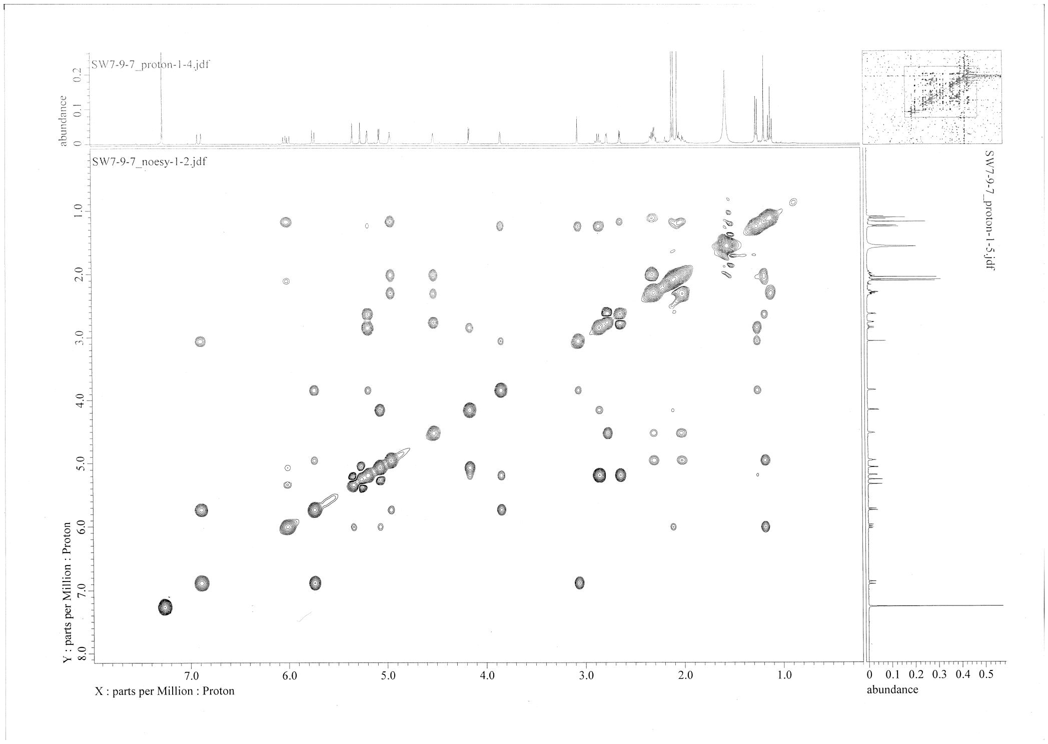

S10. NOESY spectrum of compound **1** in $CDCl_3$

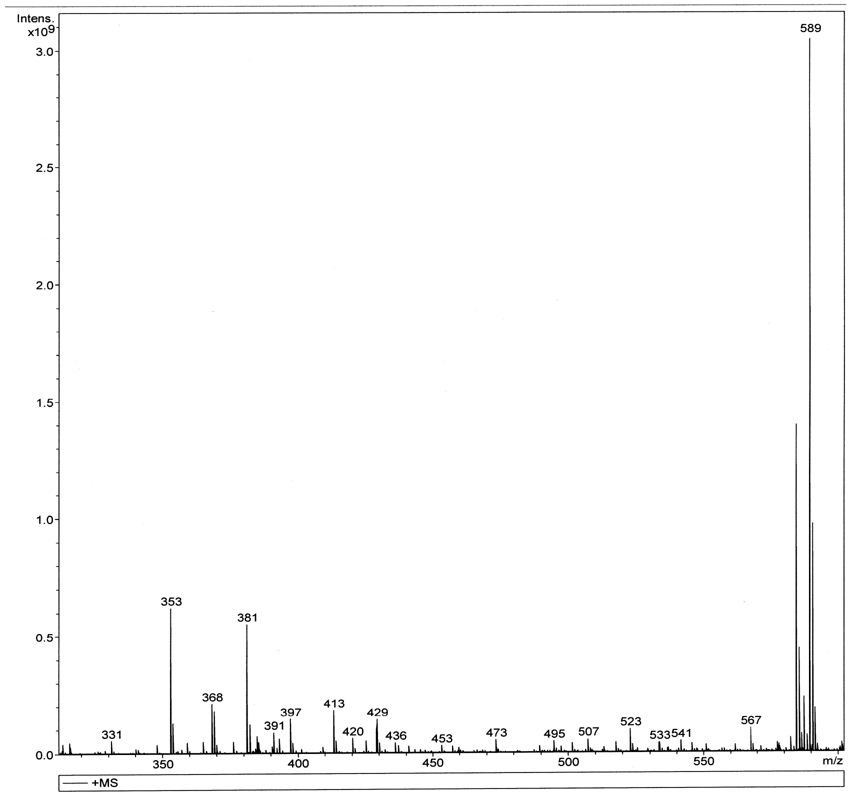

S11. ESIMS spectrum of compound **2**

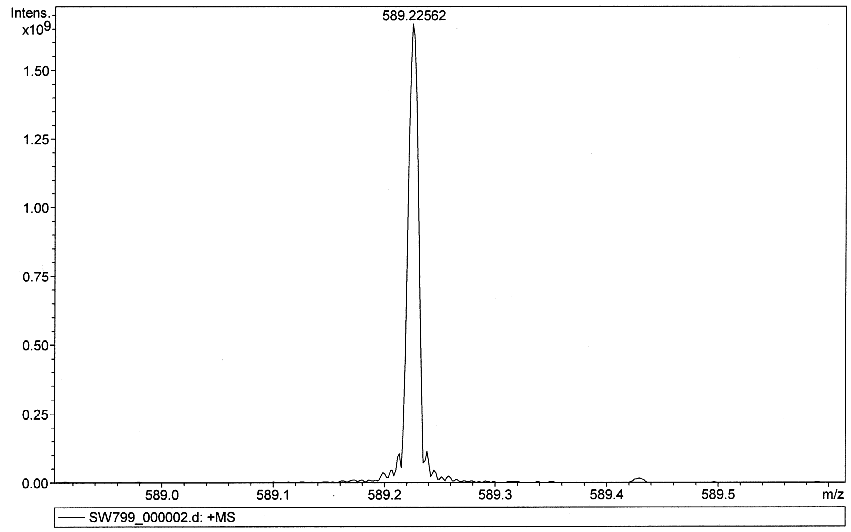

Meas. m/z	#	Formula	Score	m/z	err [mDa]	err [ppm]	mSigma	rdb	e⁻ Conf	N-Rule
589.22562	1	C 28 H 38 Na O 12	100.00	589.22555	-0.08	-0.13	8.2	9.5	even	ok

S12. HRESIMS spectrum of compound **2**

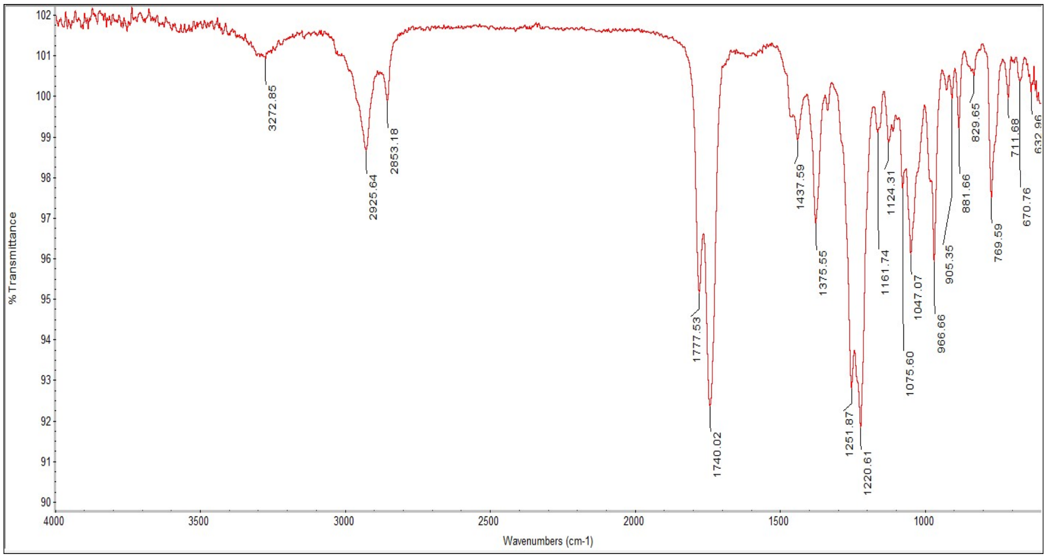

S13. IR spectrum of compound **2**

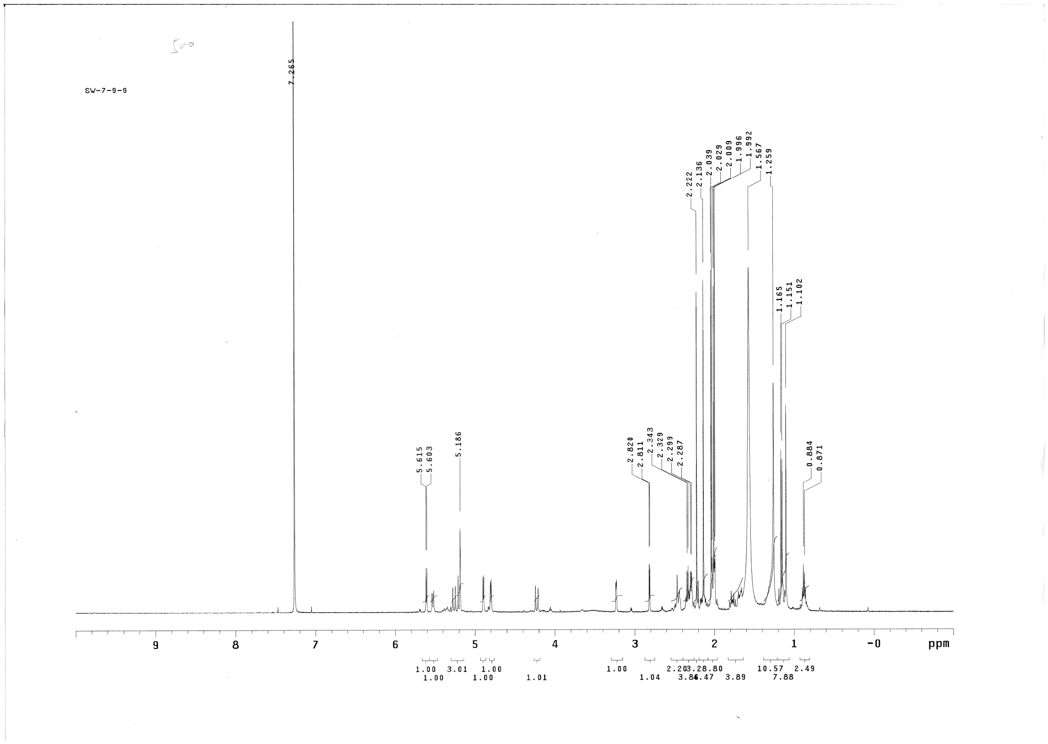

S14. ^{1}H NMR spectrum (500 MHz) of compound **2** in $CDCl_3$

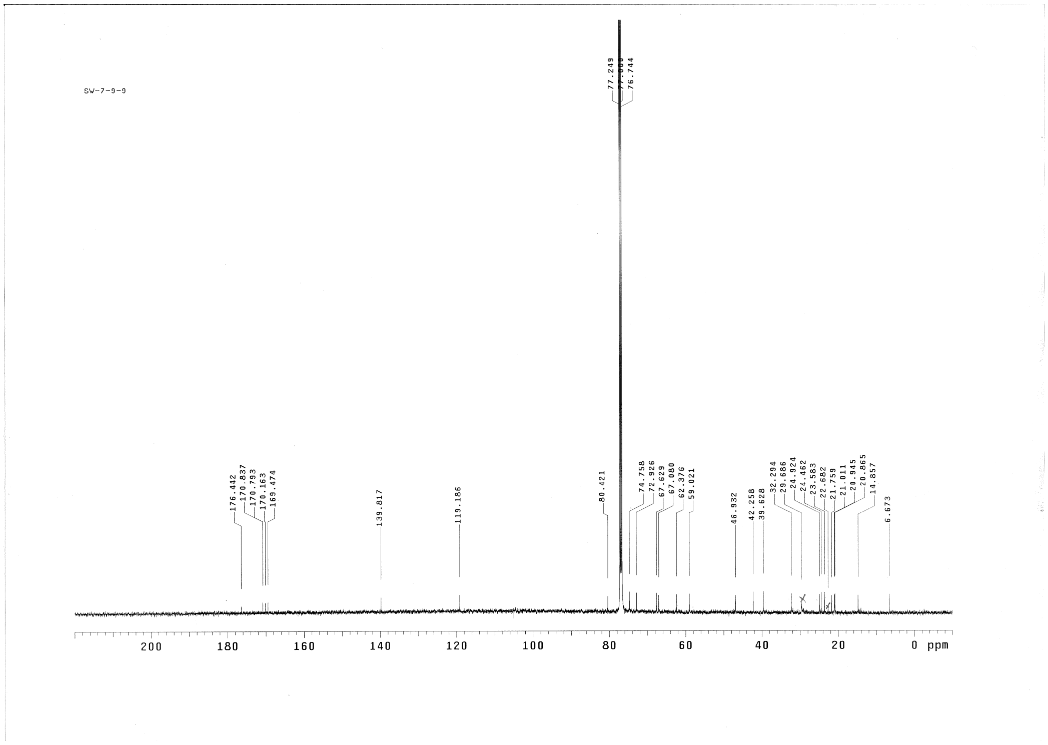

S15. ^{13}C NMR spectrum (125 MHz) of compound **2** in $CDCl_3$

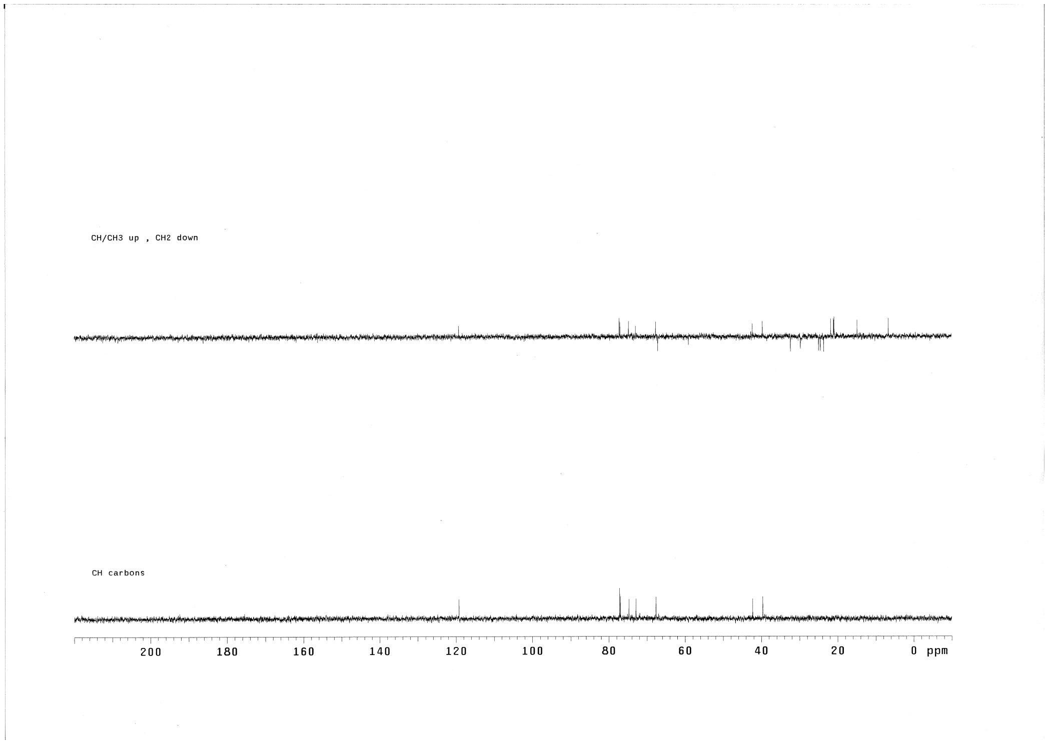

S16. DEPT spectrum of compound **2** in $CDCl_3$

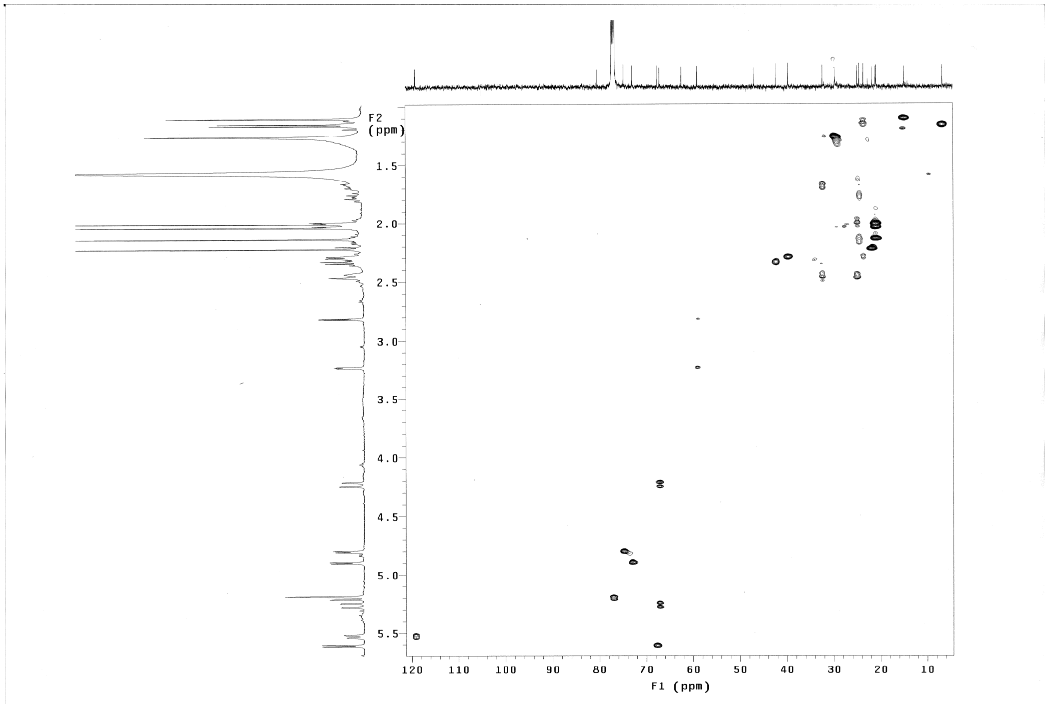

S17. HSQC spectrum of compound **2** in $CDCl_3$

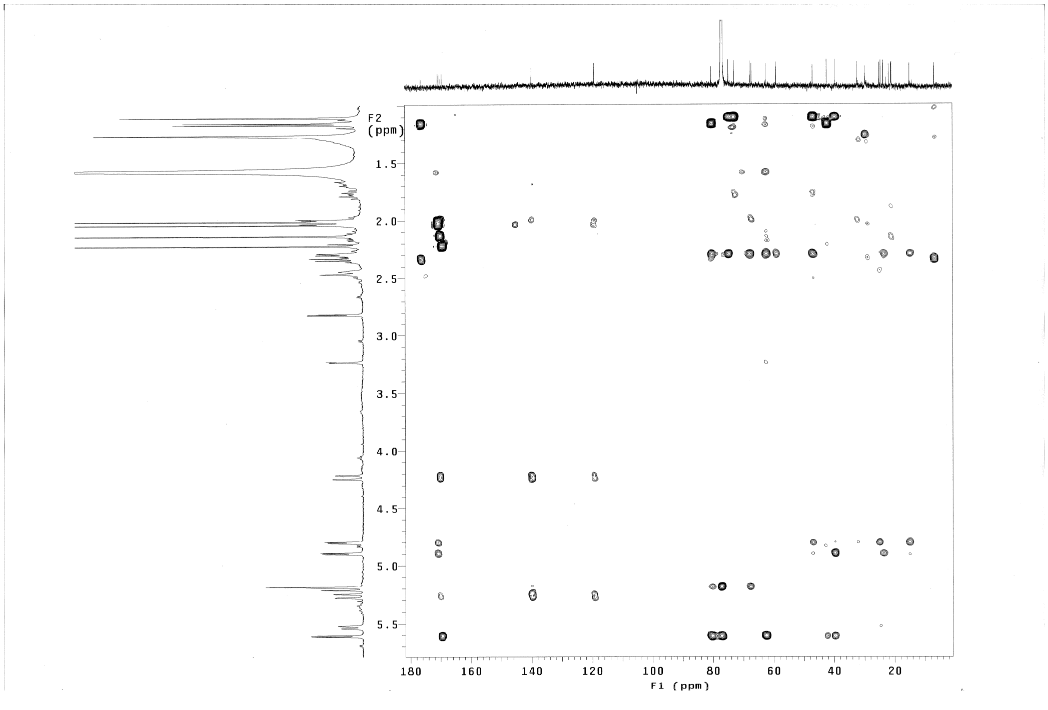

S18. HMBC spectrum of compound **2** in $CDCl_3$

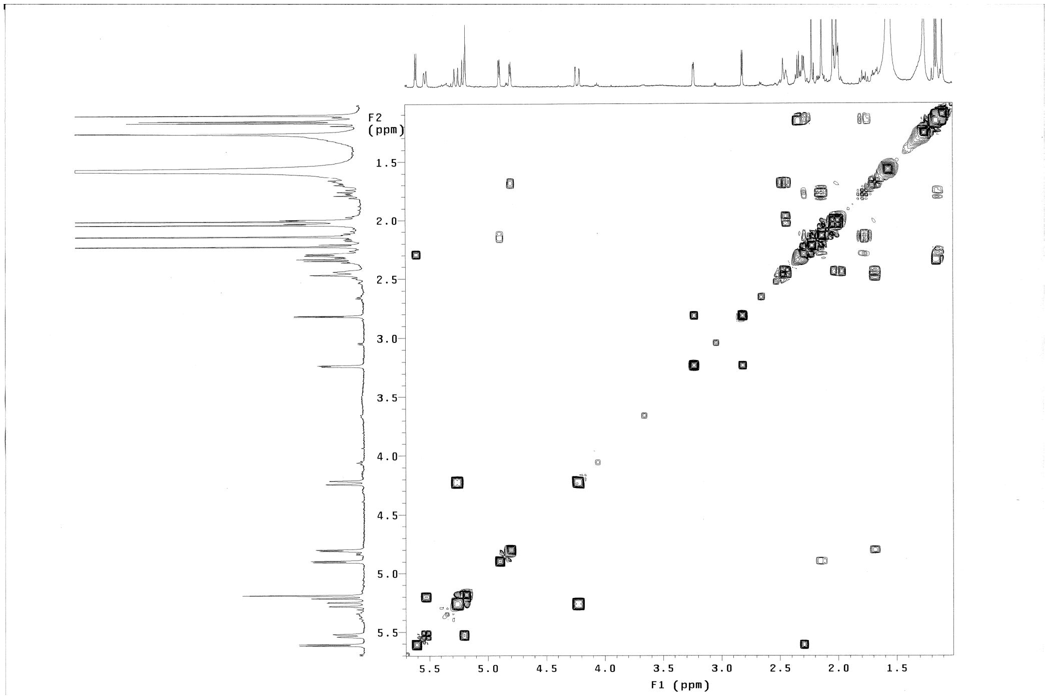

S19. COSY spectrum of compound **2** in $CDCl_3$

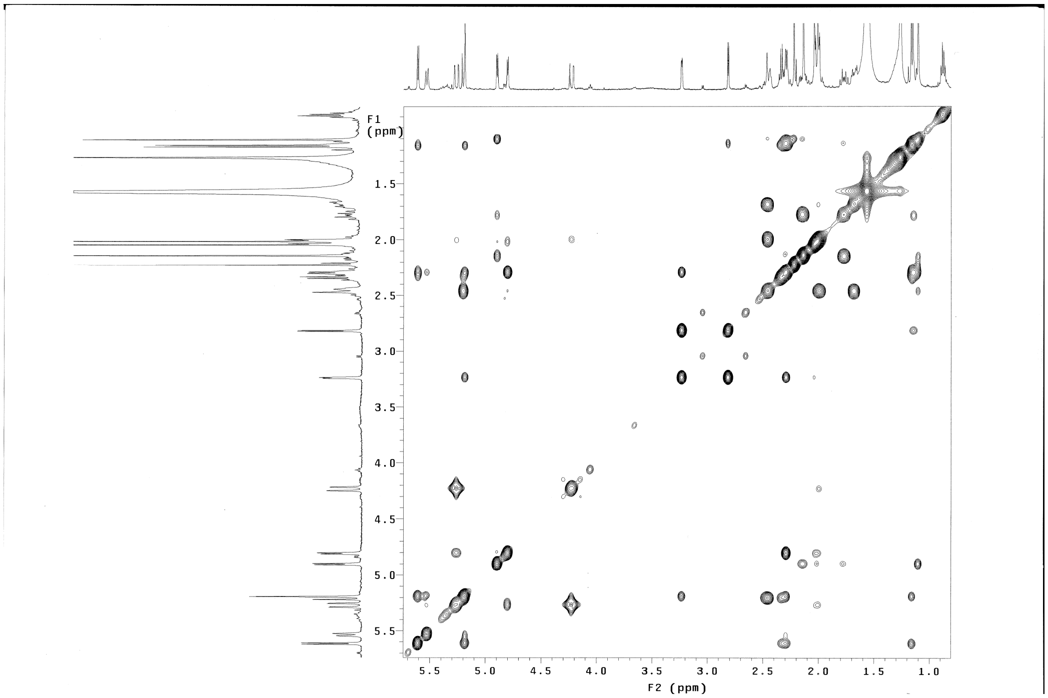

S20. NOESY spectrum of compound **2** in $CDCl_3$

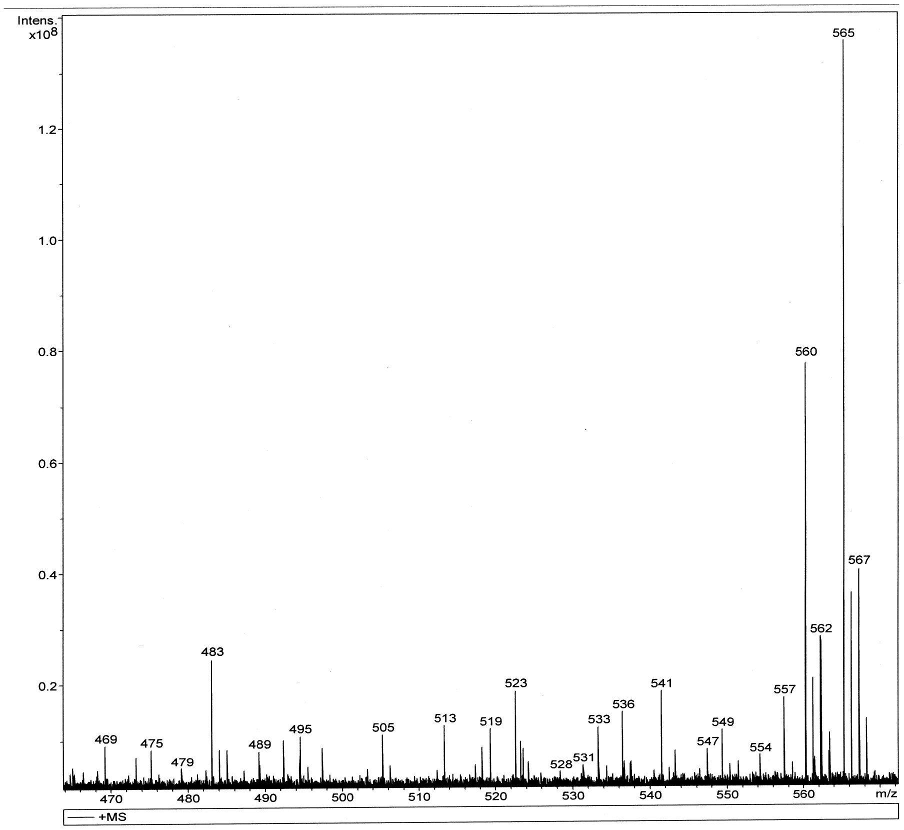

S21. ESIMS spectrum of compound **3**

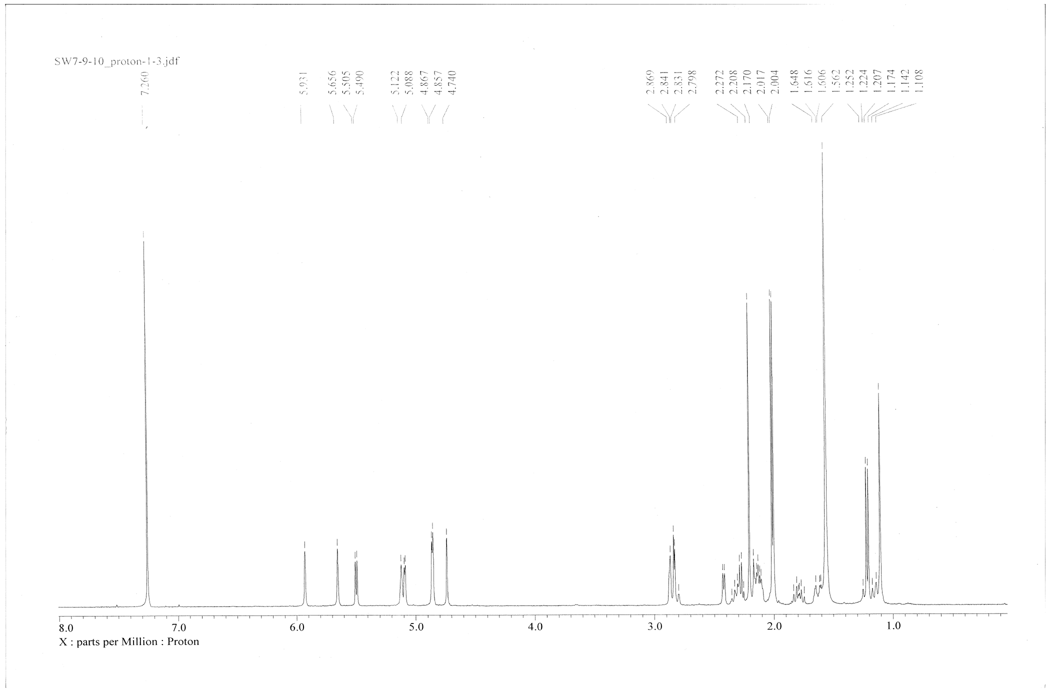

S22. ^{1}H NMR spectrum (400 MHz) of compound **3** in $CDCl_3$

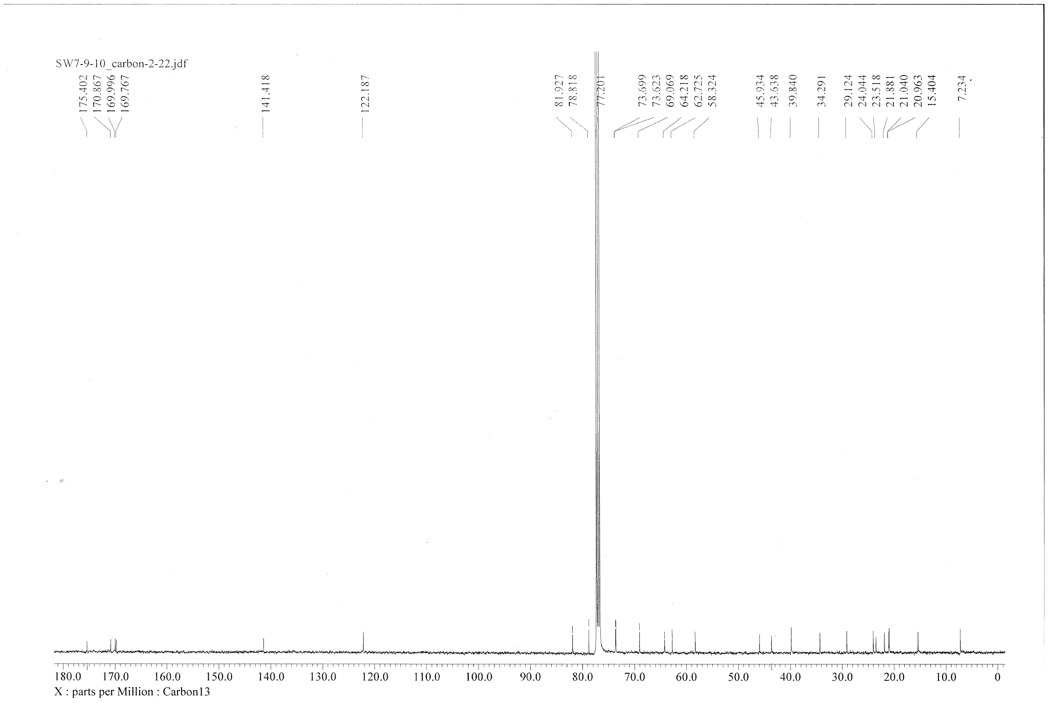

S23. ^{13}C NMR spectrum (100 MHz) of compound **3** in $CDCl_3$

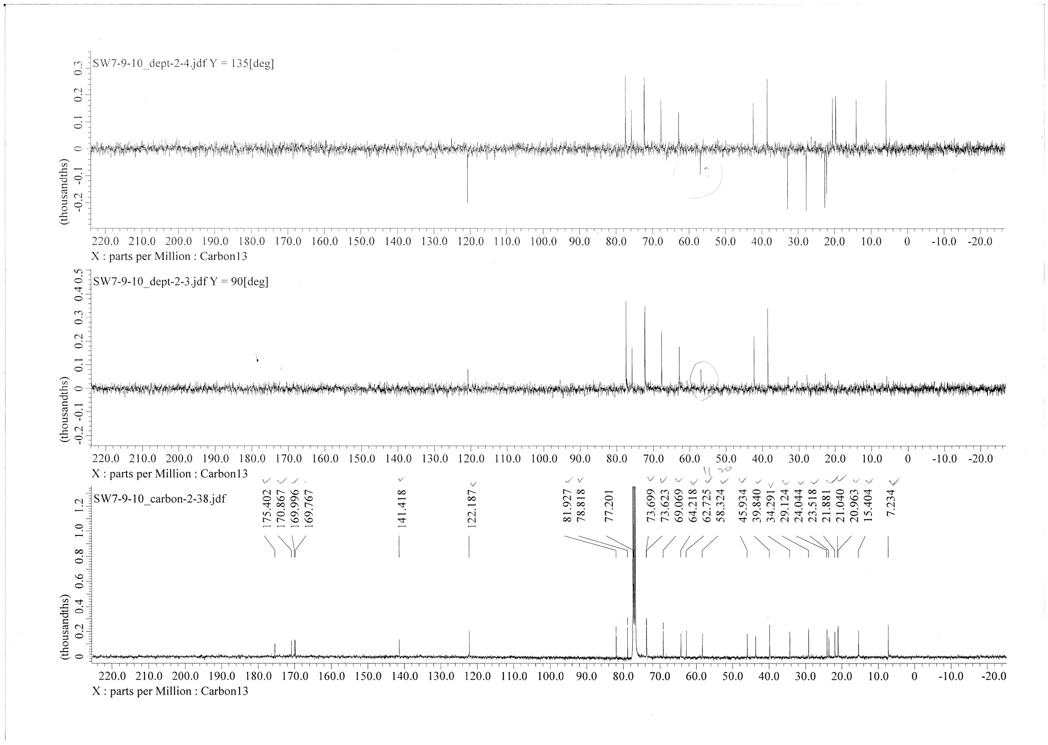

S24. DEPT spectrum of compound **3** in $CDCl_3$

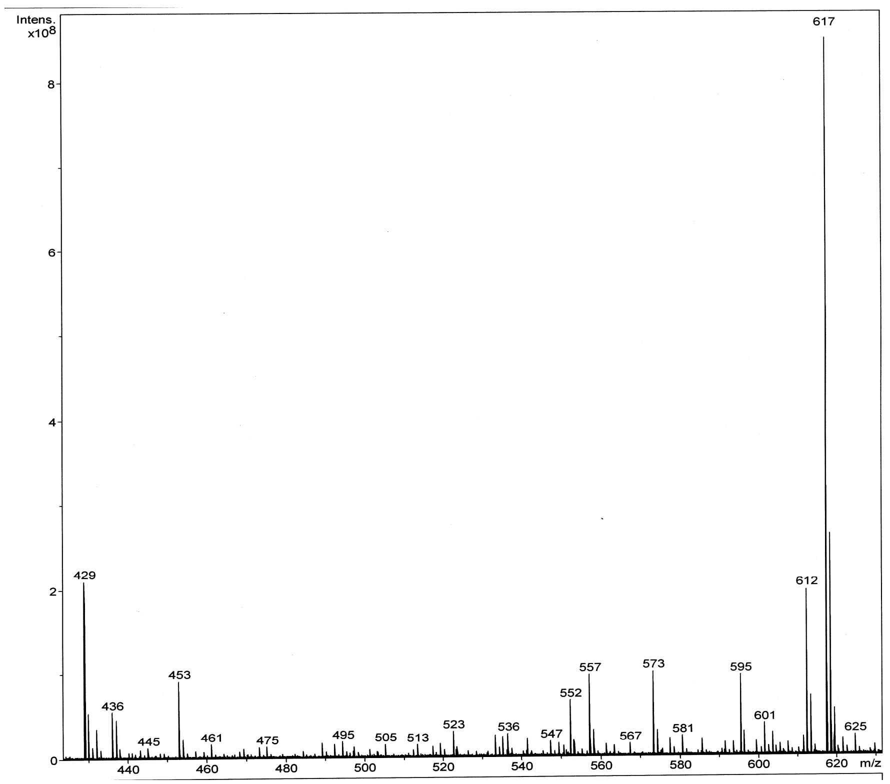

S25. ESIMS spectrum of compound **4**

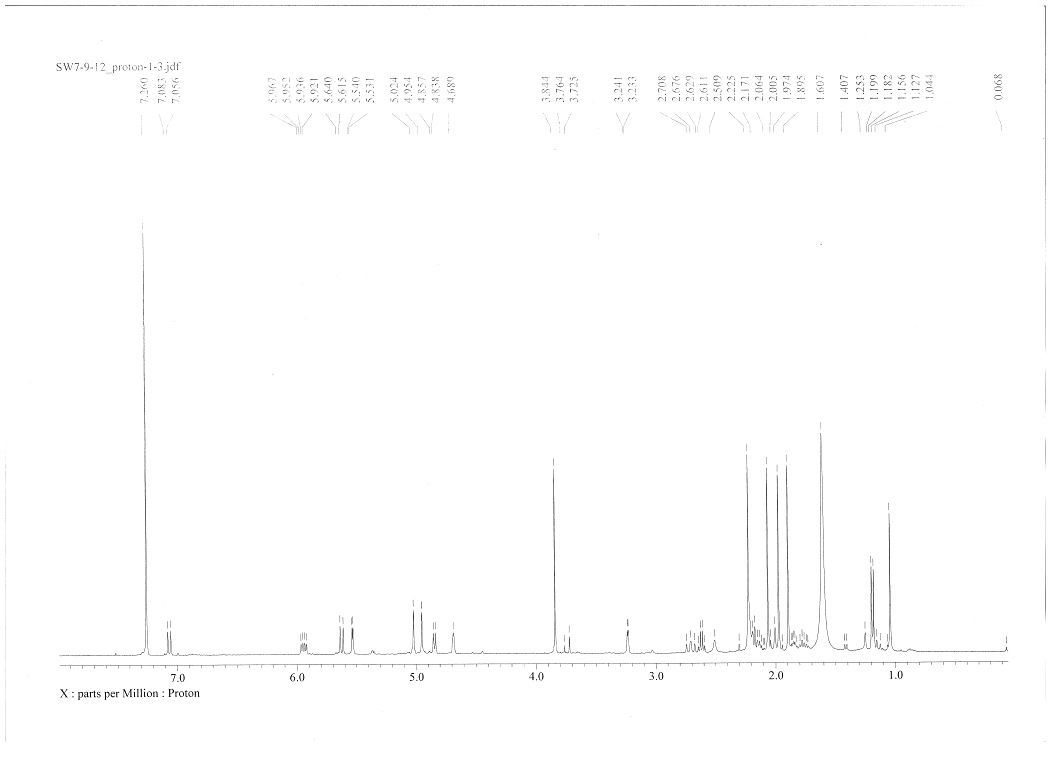

S26. ^{1}H NMR spectrum (400 MHz) of compound **4** in $CDCl_3$

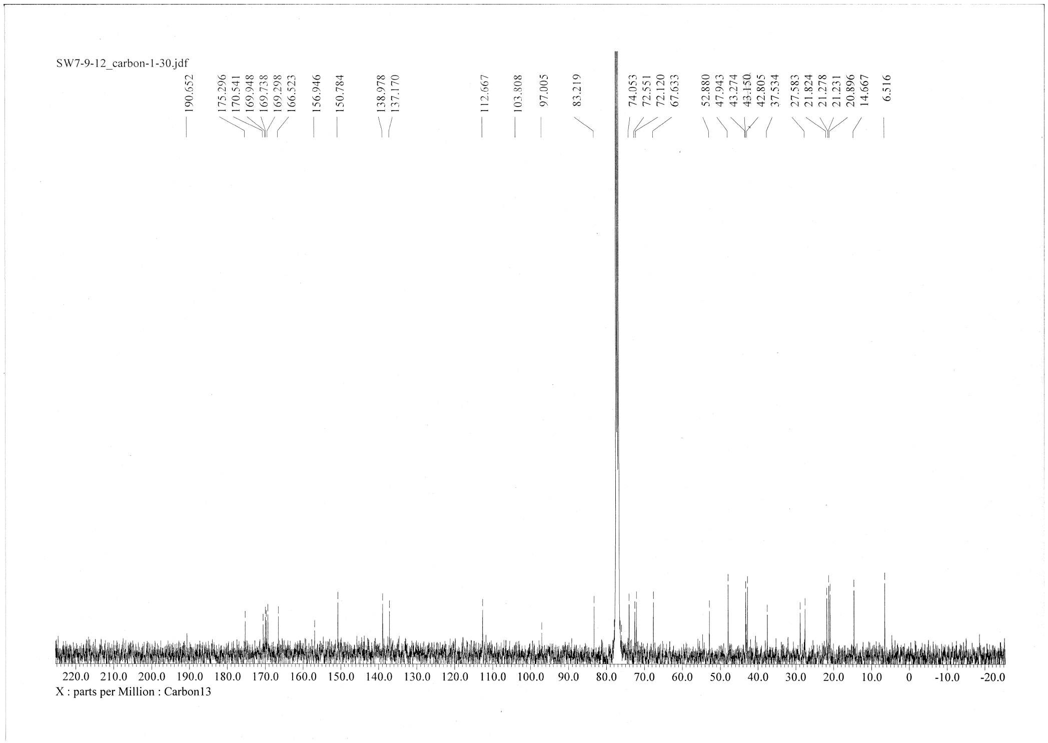

S27. ^{13}C NMR spectrum (100 MHz) of compound **4** in $CDCl_3$

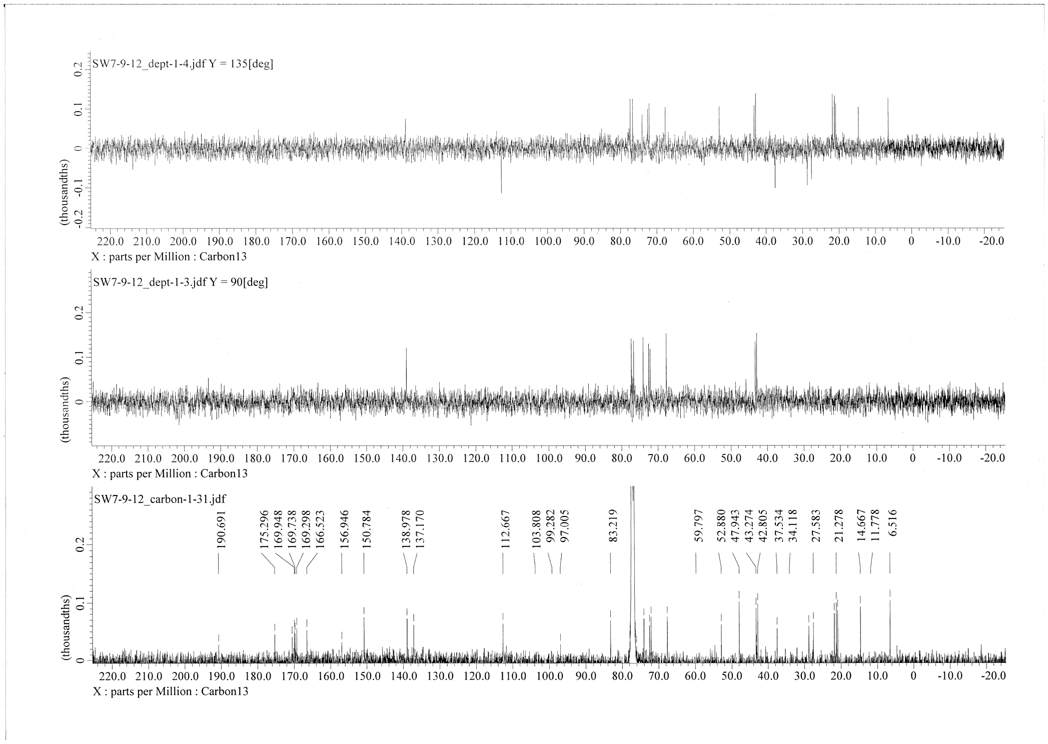

S28. DEPT spectrum of compound **4** in $CDCl_3$
